# Fatty acid- and retinol-binding protein 6 does not control worm fatty acid content in *Caenorhabditis elegans* but might play a role in *Haemonchus contortus* parasitism

**DOI:** 10.1186/s13071-023-05836-8

**Published:** 2023-07-10

**Authors:** Fei Wu, Haidian Wei, Xueqiu Chen, Zhendong Du, Yan Huang, Hengzhi Shi, Yi Yang, Aifang Du, Guangxu Ma

**Affiliations:** 1 College of Animal Sciences, Zhejiang Provincial Key Laboratory of Preventive Veterinary Medicine, Institute of Preventive Veterinary Medicine, Zhejiang University, Hangzhou, 310058 China; 2 Guangxi Key Laboratory of Veterinary Biotechnology, Guangxi Veterinary Research Institute, Nanning, 530004 China

**Keywords:** *Caenorhabditis elegans*, *Haemonchus contortus*, Fatty acid- and retinol-binding protein, FAR-6, Nematode parasitism

## Abstract

**Background:**

Nematodes have lost the ability to synthesise necessary lipids de novo and have complementally evolved the capacity to acquire fatty acids and their derivatives from a diet or host animal. Nematode-specific fatty acid- and retinol-binding protein (FAR) family is one approach that facilitates lipid acquisition, representing an Achilles heel and potential target against roundworms of socioeconomic significance. However, little is known about their detailed functional roles in either free-living or parasitic nematodes.

**Methods:**

A genome-wide identification and curation were performed to screen the FAR family members of *Haemonchus contortus*. Their transcription patterns in worms were also analysed to identify the targets. Ligand binding assay and molecular docking were conducted to verify the fatty acid binding activities of FAR proteins of interest. RNA interference (RNAi) and heterologous expression (rescuing) experiments were designed to explore the potential roles of the selected FAR protein in nematodes. Localisation of the protein was shown in sections of paraffin-embedded worms after an immunohistochemistry (IHC) assay.

**Results:**

Here, an orthologue of *far-6* in the model organism *Caenorhabditis elegans* (*Ce-far-6*) was functionally characterised in a parasitic nematode, *H. contortus* (*Hc-far-6*). It is demonstrated that knockdown of *Ce-far-6* gene did not affect worm fat content, reproduction, or lifespan, but decreased worm body length at an early life stage of *C. elegans*. In particular, the *Ce-far-6* mutant associated phenotype was completely rescued by *Hc-far-6*, suggesting a conserved functional role. Surprisingly, there were distinct tissue expression patterns of FAR-6 in the free-living *C. elegans* and parasitic *H. contortus*. High transcriptional level of *Hc-far-6* and dominant expression of FAR-6 in the intestine of the parasitic stage of *H. contortus* link this gene/protein to nematode parasitism.

**Conclusions:**

These findings substantially enhance our understanding of *far* genes and the associated lipid biology of this important parasitic nematode at a molecular level, and the approaches established are readily applicable to the studies of *far* genes in a broad range of parasites.

**Graphical Abstract:**

**Figure:**
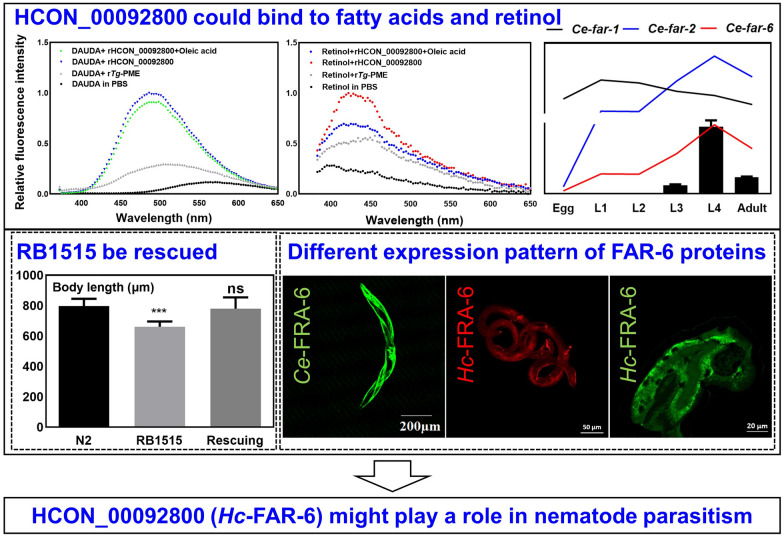

**Supplementary Information:**

The online version contains supplementary material available at 10.1186/s13071-023-05836-8.

## Background

Parasitic nematodes are major pathogens of humans, animals (including pets and livestock), and plants (including trees and crops), causing global socioeconomic losses that have been estimated at tens of billions of dollars annually [1–5]. Using a mass administration of anthelmintics including macrolides, benzimidazoles, and imidazolothiazoles [6–8], some key parasitic and neglected tropical diseases (e.g. dracunculiasis, lymphatic filariasis, and onchocerciasis) have been eliminated, and nematode infection of animals has been effectively controlled in some countries and regions. However, due to the wide use of anthelmintics, the issue of drug resistance has been reported in a wide range of parasitic nematodes, particularly in nematodes of livestock, and the worldwide and multiple resistance has been a concern for over 70 years [9–13]. It is imperative to develop new and innovative interventions, preferably chemicals or vaccines that target nematode-specific molecules to suppress nematode infections.

Lipids are molecules involved in a variety of biological processes and are essential for all life forms. For instance, fatty acids are required for collagen synthesis, cuticle construction, and nematode development, and retinol or retinoic acids are crucial in cell signalling, tissue differentiation, and immune-related responses in parasitic nematodes [14–18]. Remarkably, it is thought that both free-living and parasitic nematodes have lost the genes associated with de novo lipid biosynthesis [19, 20]. Fatty acid- and retinol-binding proteins (FARs), venom allergen-like proteins (VALs), and nematode polyprotein antigens (NPAs) are three kinds of lipid-binding proteins facilitating nematode sequester lipids and related precursors from an external source [21–23]. In the model organism *Caenorhabditis elegans* (a free-living nematode), there are 10 genes (i.e. *far-1*, -*2*, -*3*, *-4*, *-5*, *-6*, *-7*, *-8*, -*9*, and *perm-5*) predicted to encode proteins that enable lipid (fatty acid and retinol) binding activity. Genome-wide RNA interference has linked phenotypic changes to certain *far* genes in the free-living *C. elegans*, including but not limited to “dumpy in *far-1*” (WBRNAi00008600), “fat content increased in *far-2*” (WBRNAi00113974), and “embryonic lethal in *perm-5*” (WBRNAi00008557) [24, 25]. Thus, although most *far* genes (including *far-1* and *far-2*) are predicted paralogues in *C. elegans*, they appear to exhibit functional divergence in multiple biological processes of this free-living nematode.

Importantly, FARs are known as nematode-specific lipid-binding proteins with no orthologues predicted in other organisms [23, 26] and have been reported to play crucial roles in nematode infection, development, and reproduction [27–31], representing an Achilles heel and potential targets against nematodes of socioeconomic significance. However, although genome-wide identification of *far* genes has been performed in select parasitic nematodes [23, 26, 32], there is a lack of systematic nomenclature rules and comprehensive functional information for *far* genes in these pathogens, particularly at the molecular level. This is mainly due to the limited functional genomics and lipid biology of parasitic nematodes, hindering the screening of *far* genes for potential novel intervention targets.

In this work, by conducting a comparative study with the model organism *C. elegans*, *far* genes in *Haemonchus contortus* (barber’s pole worm) have been identified, manually curated, and screened for molecules that likely play a role in the key developmental stage, with a particular focus on *far* genes that are highly transcribed in the pathogenic stages of this important parasite.

## Materials and methods

### Experimental animals, nematodes, and cell line

Hu sheep (*n* = 2) were used to maintain the parasitic stages of *H. contortus* (barber’s pole worm) *in vivo*. Kunming mice (*n* = 5) were used to produce polyclonal antibodies against the nematode-specific FAR protein.

The free-living nematode *C. elegans* (wild-type N2 strain and mutant-type RB1515 strain) was obtained from the Caenorhabditis Genetics Center (CGC) and maintained following the manufacturer’s instructions. Parasitic nematode *H. contortus* (ZJ isolate) was experimentally maintained in Hu sheep and the eggs, first- (L1s), second- (L2s), third- (L3s), and fourth-stage larvae (L4s), and the adult worms were collected as described previously [33, 34]. The human embryonic kidney 293 T (HEK 293 T) cell line (Beyotime, Shanghai) was maintained following the manufacturer’s instructions.

### Genome-wide identification and curation

Genes encoding proteins of the nematode-specific FAR family (also known as the *Gp*-FAR-1 family; PF05823) were identified in *C. elegans* and *H. contortus* based on genomic datasets available at WormBase ParaSite (Version: WBPS15). Candidates were integrated with previously reported FAR sequences in *H. contortus* [26, 32], using a manual gene family curation approach [35]. Orthologues (1-to-1, 1-to-many, or many-to-many) between *C. elegans* and *H. contortus* were predicted using the Compara Gene Tree (International Helminth Genomes Consortium, 2019). Amino acid sequences deduced from the FAR coding genes in both *C. elegans* and *H. contortus* were aligned using EMBOSS Needle or Clustal W, which were then subjected to a phylogenetic analysis using the Maximum-like model in MEGA X v.10.1.8 [36].

### Quantitative reverse transcription PCR (qRT-PCR) analysis

Total RNA was isolated from *H. contortus* eggs, L1s, L2s, L3s, L4s, or adult worms using Trizol reagent (Invitrogen, China) and reverse transcribed into the first-strand cDNA using ReverTra Ace-α (TOYOBO, China). A mixture (20 μl) of AceQ Universal SYBR qPCR Master Mix (Vazyme Biotechnology Ltd., Nanjing, China) was prepared following the manufacturer's instructions and incubated in a LightCycler 480 Instrument II (Roche Diagnostics Ltd., Basel, Switzerland). Relative transcriptional levels of HCON_00092800 among developmental stages of *H. contortus* were calculated using a 2^−ΔΔCt^ method [37], with the β-tubulin gene used as an internal reference gene. Three technical replicates were included in this experiment. Primers for amplification and quantification were included in Additional file 1: Table S1.

### Molecular cloning and expression

The coding sequence of a certain *far* gene in *H. contortus* (HCON_00092800; previously known as HCOI_00909000, HCOI_00908900, and HCOI_01356800) was PCR amplified, sequenced, and inserted into the pET-30a vector via *Sac*I and *Kpn*I restriction sites. The constructed expression vector was transformed into BL21 (DE3) *Escherichia coli* competent cells. Isopropyl β-D-thiogalactoside (1 mM) was used to induce the expression of His tag-fused FAR in the transformed bacteria. Recombinant HCON_00092800 protein was purified using a Ni–NTA agarose column (Qiagen, Shanghai, China). The coding sequence was also inserted into the pEGFP-C2 vector, which was then transfected into HEK 293 T cells using polyethylenimine (PEI, Linear). After 48 h, transfected cells were stained with 4ʹ,6-diamidino-2-phenylindole for 15 min at 37 °C, followed by washing in phosphate-buffered saline (PBS) and visualised under the LSM 780 fluorescent microscope (Zeiss, Germany). Primers for coding sequence amplification and protein expression are included in Additional file 1: Table S1.

### Structural modelling and molecular docking

Protein structures of FAR-2 (UniProt P34383) and FAR-6 of *C. elegans* (UniProt Q9XUB7) were retrieved from AlphaFold Protein Structure Database [38]. Protein structure of HCON_00092800 (without signal peptide) of *H. contortus* was modelled using AlphaFold2 v. 2.1.0 [39]. Models were compared and visualised using UCSF ChimeraX v.1.0 [40]. Molecular docking of fatty acids with HCON_00092800 was performed using the ClusPro server [41, 42].

### Ligand-binding assay

By exploiting the molecular feature that the absorbance peaks of 11-(dansylamino) undecanoic acid (DAUDA) and retinol in free or protein-bound states are different [43], the binding activity of recombinant HCON_00092800 protein with fluorescent DAUDA (Cayman Chemical, Ann Arbor, MI, USA) and retinol (Sigma-Aldrich, St. Louis, MO, USA) as well as non-fluorescent oleic acid (Sigma-Aldrich, St. Louis, USA) was analysed on a fluorescence spectrometer Synergy H1 (Bio-Tek Instruments, Inc., Vermont, USA) using an established method [44]. In brief, DAUDA (10 μM) or retinol (10 μM) was mixed with the recombinant HCON_00092800 protein (3 μM) in PBS and incubated at 37 °C for 15 min; oleic acid (10 μM) was used as a competing ligand; ligand, recombinant pectin methylesterase of *Toxoplasma gondii* (r*Tg*-PME, a protein that does not bind fatty acids or retinol), and PBS were used as a positive, irrelevant, and negative control, respectively. The fluorescence of DAUDA was excited by a laser at 345-nm wavelength and retinol at 350-nm wavelength. The relative fluorescence intensity of the ligand in each mixture was measured by spectrum scanning in triplicates.

### RNA interference (RNAi)

A feeding method was used to conduct homologous or heterologous RNAi of *far* genes in *C. elegans* as described previously [45]. In brief, the entire coding sequence of *Ce-far-6* or HCON_00092800 was inserted into the L4440 plasmid. The recombinant plasmid was transformed into *E. coli* HT115 (DE3) strain to express specific dsRNAs. Bacteria transformed with empty vector and bacteria expressing dsRNA targeting the *cry1Ac* gene of *Bacillus thuringiensis* were used as negative and irrelevant control, respectively. RNAi experiments were conducted in triplicates. Gene knockdown analysis was performed using qRT-PCR, based on a 2^−ΔΔCt^ method [37] at 24, 48, or 60 h post bacterial feeding. Primers used for RNAi and gene knockdown analysis were included in Additional file 1: Table S1.

### Lipid staining and quantification

Fat content in RNAi-treated and -untreated *C. elegans* was determined by staining and measuring the intensity of fatty acid colorant Oil red O (Sigma-Aldrich) using an established method [46]. In brief, worms were collected, washed three times in PBS, and fixed in 2 × MRWB (160 mM KCl, 40 mM NaCl, 14 mM Na_2_EGTA, 1 mM spermidine-HCl, 0.4 mM spermine, 30 mM Na-PIPES, 0.2% β-mercaptoethanol) containing 2% paraformaldehyde at room temperature for 1 h and then incubated in 60% isopropanol at room temperature for 15 min. The treated worms were dyed in Oil red O reagent at room temperature overnight. After washing, worms (*n* = 20) were observed and imaged under an LSM 780 confocal fluorescence microscopy (Zeiss, Germany). ImageJ software (NIH Image, Bethesda, MD, USA) was used to quantify the signal intensity of Oil red O staining [47].

### Development and survival assay

Body length and survival rate of RNAi-treated and -untreated *C. elegans* were measured as described previously [48]. In brief, the average length of L4s and adult worms was determined by measuring 20 larvae/adult worms under a dissecting microscope 24 h and 48 h after RNAi [49]; the survival curve of worms was determined by counting and calculating the ratio of live worms from day 1 to day 25 after RNAi, with the number of eggs produced by the treated and untreated worms counted during the entire experiment [50].

### Heterologous expression and rescuing

Nuclear DNA was isolated from the adult worms of *C. elegans* and *H. contortus* using a TIANamp Genomic DNA Kit (TIANGEN Biotech Co., Ltd., Beijing, China) according to the manufacturer's instructions. Each promoter sequence for *Ce-far-6* and HCON_00092800 was obtained from the genomic DNA sample using primer sets amplifying about 2000 bp upstream gene locus genomic DNA. Each of the PCR products was cloned into the pPD95.77 vector to construct pPD95.77-*Ce-far-6*-prom and pPD95.77-HCON_00092800-prom plasmids. The recombinant plasmid was microinjected into the gonad of the wildtype N2 strain as previously described [51]. Expression of *Ce*-FAR-6 or HCON_00092800 protein in microinjected worms was observed and imaged by confocal fluorescence microscopy (LSM 780, Zeiss). Primers used for promoter sequence amplification and heterologous expression were included in Additional file 1: Table S1.

*Ce-far-6* deficiency recusing was conducted in a *Ce-far-6* mutant (RB1515) strain of *C. elegans*. In brief, the overexpression plasmid (*Ce*P-pPD95.77-HCON_00092800) was constructed by inserting the coding sequence of HCON_00092800 into the parental vector pPD95.77-*Ce-far-6*-prom (at the C-terminal of *Ce-far-6* promoter and N-terminal of GFP). Recombinant plasmids (100 μg/ml) were microinjected into the gonad of RB1515 young adults. Body length, number of progeny, and fatty acid content of N2 (control), and RB1515 and HCON_00092800 rescuing RB1515 *C. elegans* were measured as described above.

### Lipid extraction and gas chromatography assays

Fatty acids were isolated from wildtype (N2), RB1515, and rescued *C. elegans* using a well-established protocol [52]. The content and composition of worm fatty acids were determined using a gas chromatography method as described previously [53]. In brief, fatty acid samples were loaded into an Agilent 7890A gas chromatograph (Agilent Technologies Inc., California, USA) equipped with a DB-23 column (30 m × 0.32 mm × 0.25 µm, Agilent). Fatty acid methyl esters (including C6:0, C8:0, C10:0, C12:0, C14:0, C16:0, C16:1, C18:0, C18:1, C18:2, C18:3, C20:0, C20:1, C22:0, and C24:0) (Nu-Chek Prep, Inc., Elysian, MN, USA) were used as quantification standards for fatty-acid methyl esters. The relative content of fatty acid methyl esters in N2, RB1515 and HCON_00092800 rescued worms was determined with reference to the abundance of fatty acid methyl esters including chains from C8:0 to C24:0 (GLC 17A; Nu-Chek Prep, Inc.)

### Polyclonal antibodies production

Polyclonal antibodies against the recombinant HCON_00092800 protein were produced in Kunming mice (*n* = 5) using a well-established protocol [33, 54]. In brief, the purified recombinant protein (50 µg per mouse) was mixed with Freund’s complete adjuvant (1:1) and used for the initial immunisation of mice by subcutaneous injection. After 2 weeks, the recombinant protein (25 µg per mouse) mixed with Freund’s incomplete adjuvant (1:1) was used for booster infections after a 2-week interval. Serum was collected from immunised (antiserum) and un-immunised (non-antiserum) mice 7 days after the final booster was administrated, and the titer of antibodies was determined by ELISA. The specificity of anti-HCON_00092800 polyclonal antibodies was tested against the recombinant HCON_00092800 protein produced in HEK 293 T cells and naïve HCON_00092800 protein isolated from the parasitic stage of *H. contortus* by a Western blot analysis, using an FDbio-Femto ECL kit (Fude Biological Technology, Hangzhou, China), and a ChemiDocTM Touch Imaging System (Bio-Rad Laboratories, Hercules, CA, USA).

### Western blot

The L4s of *H. contortus* (*n* = 50), young adult offspring of *C. elegans* (*n* = 5000) microinjected with heterologous expression plasmids, or untreated N2 strain (*n* = 5000) were washed five times with M9 buffer (42 mM Na_2_HPO_4_, 22 mM KH_2_PO_4_, 85 mM NaCl, and 1 mM MgSO_4_) and resuspended in PBS (137 mM NaCl, 2.7 mM KCl, 4.3 mM Na_2_HPO_4_, and 1.47 mM KH_2_PO_4_) containing the protease inhibitor cocktail (Beyotime). The specimens were frozen in liquid nitrogen, thawed at room temperature three times, and then crushed by a sonicator (40 kHz, pulse for 1.5 s, rest for 2 s, 10 min). The supernatant was collected by centrifugation at 12,000 g at 4 ℃ for 10 min and mixed with 5 × loading buffer (Fude Biological Technology) followed by boiling for 10 min. The processed protein samples were separated by 12% polyacrylamide gel electrophoresis and then transformed to 0.22 μm PVDF membrane (Millipore Corporation, Billerica, MA, USA). The polyclonal antibodies against HCON_00092800 were used as the primary antibodies, and a commercial Peroxidase Conjugated Goat anti-Mouse IgG (H + L) (Fude Biological Technology) was employed as the second antibody.

### Immunohistochemistry (IHC) assay

The L4s and adults of *H. contortus* were fixed in 4% (*w*/*v*) paraformaldehyde for 48 h and embedded in paraffin. Worm sections (5 μm) were prepared and processed for antigen repair and blocking and then incubated with 4ʹ,6-diamidino-2-phenylindole (Sigma-Aldrich), mouse anti-HCON_00092800 polyclonal antibodies (1:1000), and secondary antibodies (goat anti-mouse IgG; Alexa Fluo Plus 488, Thermo Fisher Scientific), successively. Non-antiserum was used as a negative control. Incubated and washed sections were observed and imaged using an LSM 780 confocal laser scanning microscope (Zeiss, Germany).

### Statistical analysis

Data analysis and graph drawing were performed using GraphPad Prism 8 (San Diego, CA, USA). Error bars are shown as mean ± standard deviation (SD) or mean ± standard error of the mean (SEM). One-way ANOVA or Student’s t-test was used for statistical analyses and *P* ≤ 0.05 was used to indicate statistical significance.

## Results

### There are 12 *far* gene homologues in the parasitic nematode *H. contortus*

**Fig. 1:**
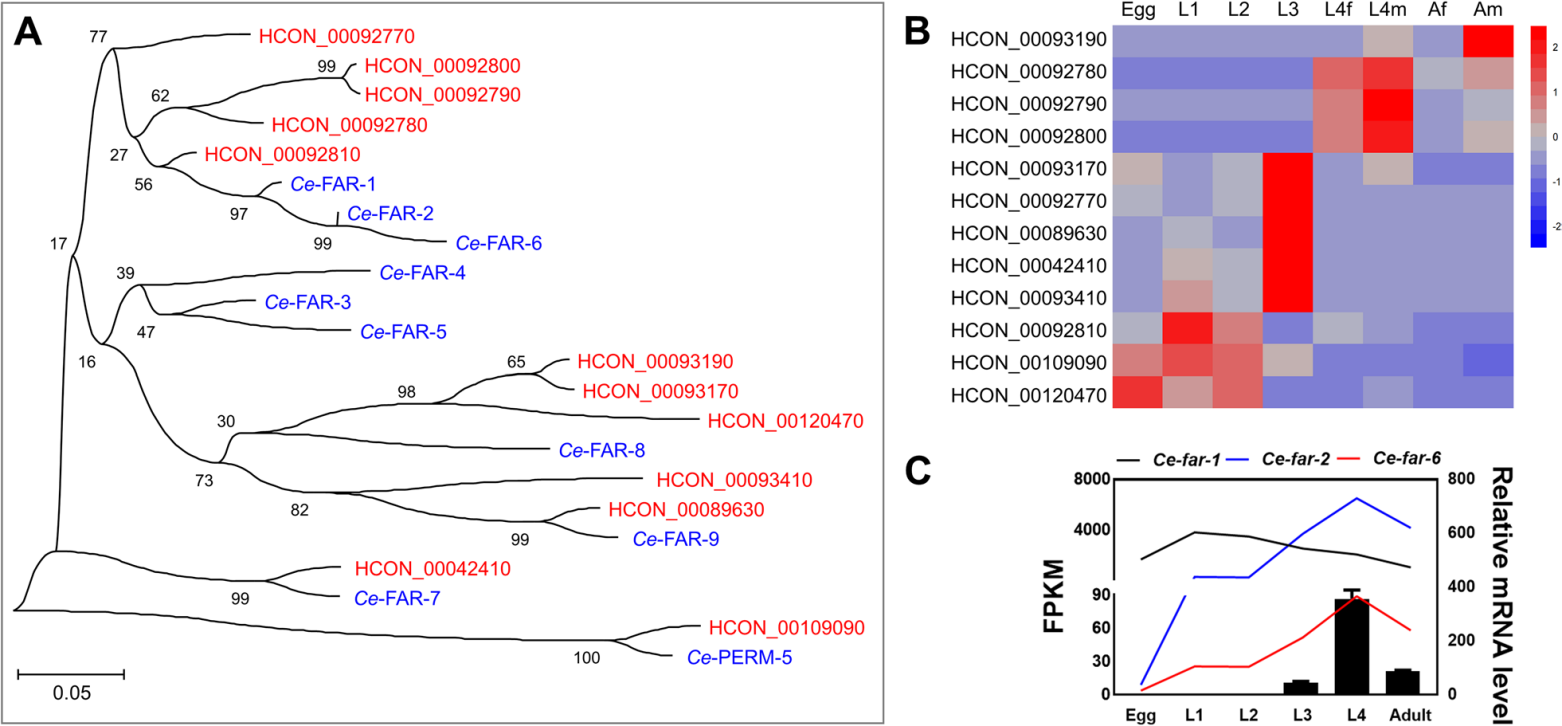
Phylogenetic tree of fatty acid- and retinol-binding protein coding genes of *Haemonchus contortus* and their transcription among developmental stages. **A** A maximum-likelihood phylogenetic tree based on amino acid sequences deduced from manually curated *HCON_00042410*, *HCON_00089630*, *HCON_00092780*, *HCON_00092790*, *HCON_00092800*, *HCON_00092810*, *HCON_00092770*, *HCON_00093170*, *HCON_00093190*, *HCON_00093410*, *HCON_00109090*, and *HCON_00120470* in *H. contortus* (WBPS15), by integrating previous work [26, 32], and FAR-1 (F02A9.2), FAR-2 (F02A9.3), FAR-3 (F15B9.1), FAR-4 (F15B9.2), FAR-5 (F15B9.3), FAR-6 (W02A2.2), FAR-7 (K01A2.2), FAR-8 (K02F3.3), FAR-9 (C07G3.10), and PERM-5 (C55C3.5) in *Caenorhabditis elegans* (WS287). **B** Transcription profiles of *far* homologues among the egg, first-(L1), second-(L2), third-(L3), and fourth-larval (L4; female and male) and adult (female and male) stages. L4f and L4m indicate sexes at the L4 stage, and Af and Am represent sexes at the adult stage. The colour scale indicates normalised fragments per kilobase per million (FPKM) of transcriptomic data [67]. **C** FPKM of *Ce-far-1*, *Ce-far-2*, and *Ce-far-6* among egg, L1, L2, L3, L4, and adult stages of *C. elegans*, and relative mRNA levels of *HCON_00092800* to *beta-tubulin* gene among egg, L1, L2, L3, L4, and adult stages of *H. contortus*. Error bars indicate the mean ± standard error of the mean (SEM)

With reference to the biological information on fatty acid-/retinol-binding protein coding genes (*far*) in the free-living model organism *C. elegans*, homologues (*n* = 12) were predicted in the blood-feeding nematode *H. contortus* using an integrative approach (Additional file 2: Table S2). Based on amino acid sequence similarity and phylogeny, 1-to-1 orthologues of *C. elegans* (*far-7* and *perm-5*) and many-to-many orthologues (*far-1*, *-2*, *-3*, *-4*, *-5*, *-6*, *-8*, and *-9*) were predicted in the parasitic *H. contortus* (Fig. 1A).

Transcriptional analysis of *far* genes in *H. contortus* showed distinct patterns among the egg, L1, L2, L3, L4, and adult stages (Fig. 1B). Specifically, five genes (*HCON_00093170*, *HCON_00092770*, *HCON_00089630*, *HCON_00042410*, and *HCON_00093410*) were highly transcribed in the infective L3 stage, three genes (*HCON_00092780*, *HCON_00092790*, and *HCON_00092800*) in the parasitic L4 stage and one gene (*HCON_00093190*) in the adult stage of *H. contortus*. Different transcriptional levels of eight *far* genes were also detected between the sexes (female and male) at either the L4 or the adult stage of this parasitic nematode. qRT-PCR analysis confirmed the transcriptional pattern for a selected gene orthologue candidate of *Ce-far-1/2/6* in *H. contortus* (i.e. *HCON_00092800*), which is in accordance with the transcriptional alterations of *Ce-far-2* and *Ce-far-6*, but not *Ce-far-1*, in *C. elegans* (Fig. 1C). Although the gene identity between *HCON_00092800* and *Ce-far-1* was not low (Additional file 3: Fig. S1), the former should play a role in *H. contortus* similar to that of *far-2* or *far-6* in the free-living *C. elegans*.

### HCON_00092800 protein binds fatty acids in silico and in vitro

**Fig. 2:**
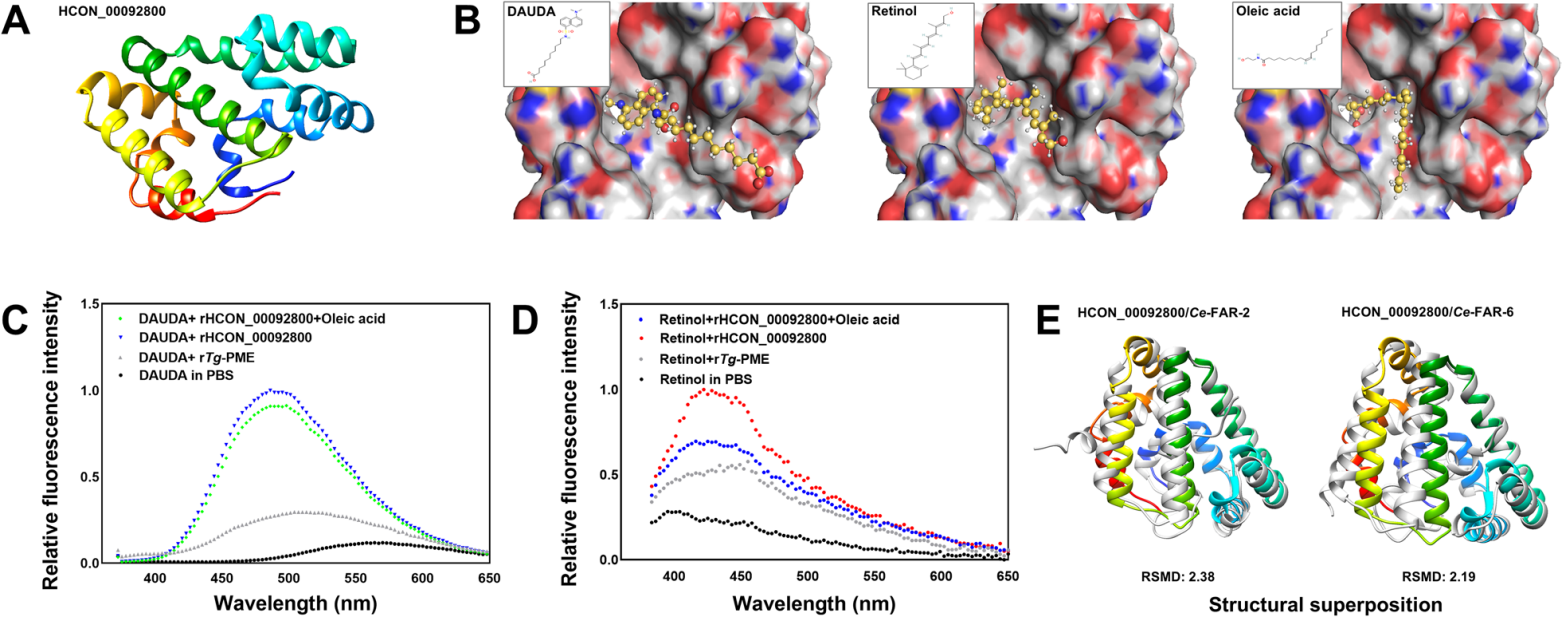
In silico molecular docking and in vitro fatty acid binding analyses for HCON_00092800 protein. **A** Three-dimensional structure of HCON_00092800 protein modelled using AlphaFold2 [39]. **B** Molecular docking of HCON_00092800 protein and DAUDA, retinol, or oleic acid, with binding free energy (ΔG) measured at − 109.81 kcal/mol, − 103.79 kcal/mol, or − 97.16 kcal/mol, respectively. **C**, **D** Relative fluorescence intensity of recombinant HCON_00092800 (rHCON_00092800) mixed with DAUDA, retinol, and/or oleic acid to that of fatty acids dissolved in phosphate-buffered saline (PBS). And r*Tg*-PME (a recombinant protein from *Toxoplasma gondii*) is employed as an irrelevant control. **E** Structural alignment and superposition of HCON_00092800 with *Ce*-FAR-2 (UniProt ID P34383) and *Ce*-FAR-6 (UniProt ID Q9XUB7)

In *C. elegans*, FAR-2 and FAR-6 are predicted to enable fatty acid binding activity and retinol-binding activity. To determine the molecular function of the protein encoded by *HCON_00092800*, the three-dimensional structure of HCON_00092800 protein was modelled (Fig. 2A), which bound DAUDA, retinol, or oleic acid in the same “pocket” in silico, with binding free energy (ΔG) predicted at − 109.81 kcal/mol, − 103.79 kcal/mol, and − 97.16 kcal/mol, respectively (Fig. 2B). An in vitro binding assay was performed to confirm these in silico findings. It was found that the absorbance peaks of fluorescent DAUDA (at 570 nm) and retinol (at 396 nm) were shifted to 486 nm and 425 nm, respectively, when mixed and incubated with recombinant HCON_00092800 in vitro (Fig. 2C, D). The fluorescence intensities of protein bound DAUDA and retinol were decreased by non-fluorescent oleic acid (Fig. 2C, D), confirming a more potent binding affinity of oleic acid (lower ΔG) than DAUDA or retinol with HCON_00092800 protein.

To better understand the structure-based fatty acid/retinol-binding activity of HCON_00092800 protein in the blood-feeding *H. contortus*, the modelled structure of this protein was compared with the structures of FAR-2 and FAR-6 of *C. elegans* (encoded by the genes exhibiting similar transcriptional profiles to HCON_00092800 in *H. contortus*). Superposition of the structures of HCON_00092800 and *Ce-*FAR-2 (root-mean-square deviation 2.38 and Q-score 0.31) or FAR-6 (2.19 and 0.22) was performed in silico (Fig. 2E), indicating similar structural-based functional roles among these molecules in both free-living and parasitic nematodes.

### RNAi activation with heterologous *HCON_00092800* does not influence fat content in *C. elegans*

**Fig. 3:**
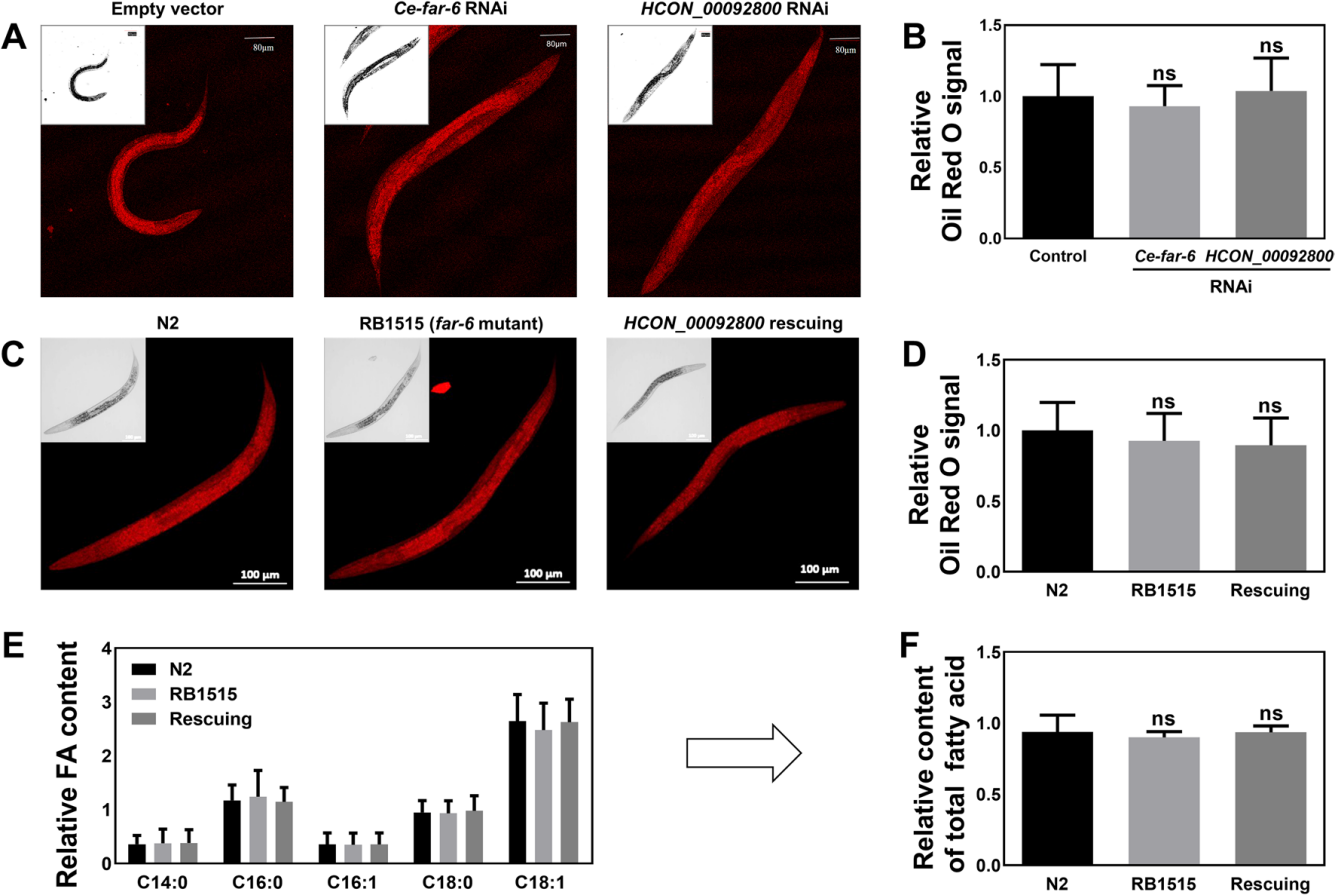
Influence of *Ce-far-6* deficiency and heterologous *HCON_00092800* RNA interference (RNAi) on fatty acid content in *Caenorhabditis elegans*. **A**, **B** Fat content in *C. elegans* after *Ce-far-6* and *HCON_00092800* sequence-mediated RNA interference treatment, determined by Oil Red staining. *cry1Ac* is used as an irrelative control. Scale bar: 80 μm. **C**, **D** Fat content in wildtype (N2), *far-6* mutant (RB1515), and HCON_00092800 rescuing *C. elegans*, determined by Oil Red staining. *cry1Ac* is used as an irrelevant control. Scale bar: 100 μm. Relative Oil Red signal is quantified using ImageJ software [47]. **E**, **F** Relative content of select (C14:0, C16:0, C16:1, C18:0, and C18:1) and total fatty acids in N2, RB1515, and HCON_00092800 rescuing worms. GLC 17A (PRIME) (fatty acid methyl esters containing chains from C8:0 to C24:0; Nu-Chek Prep, Inc.) served as inner controls. Error bars indicate mean ± standard deviation (SD); ns = not significant

In the model organism *C. elegans*, RNAi of *far-2* has been linked to an increased fat content [25]. To refine the functional annotation of the *HCON_00092800* gene in parasitic nematodes, dsRNA of the full-length transcript of this gene was used to trigger heterologous RNAi in the free-living nematode *C. elegans*. However, compared with negative (empty vector) and homologous controls (*Ce-far-6*), increased fat content was observed in neither the heterologous RNAi-treated worms nor *Ce-far-6* RNAi-treated worms (Fig. 3A, B).

To support this proposal, a *Ce-far-6* mutant and HCON_00092800 rescuing assay was performed in *C. elegans*. Compared with the wildtype (N2) strain of *C. elegans*, the *Ce-far-6* mutant (RB1515) exhibited neither significantly increased nor decreased fat content (Fig. 3C, D; Additional file 4: Fig. S2A). This result was confirmed by the relative content of select fatty acids (e.g. C14:0, C16:0, C16:1, C18:0 and C18:1) and the relative content of total fatty acids between N2 and RB1515 strains of *C. elegans* (Fig. 3E, F). Heterologous expression of HCON_00092800 protein did not influence the fat content in RB1515 worms (Fig. 3C–F).

Therefore, although there are high sequence and structural similarities between far-2 and far-6 in *C. elegans* (Fig. 2E), *far-6* might play a different role from *far-2* in *C. elegans*, and HCON_00092800 is probably a functional orthologue of *Ce-far-6* (Figs. 1, 2, 3).

### *HCON_00092800* dsRNA mediates gene knockdown of *Ce-far-6* in *C. elegans*

**Fig. 4:**
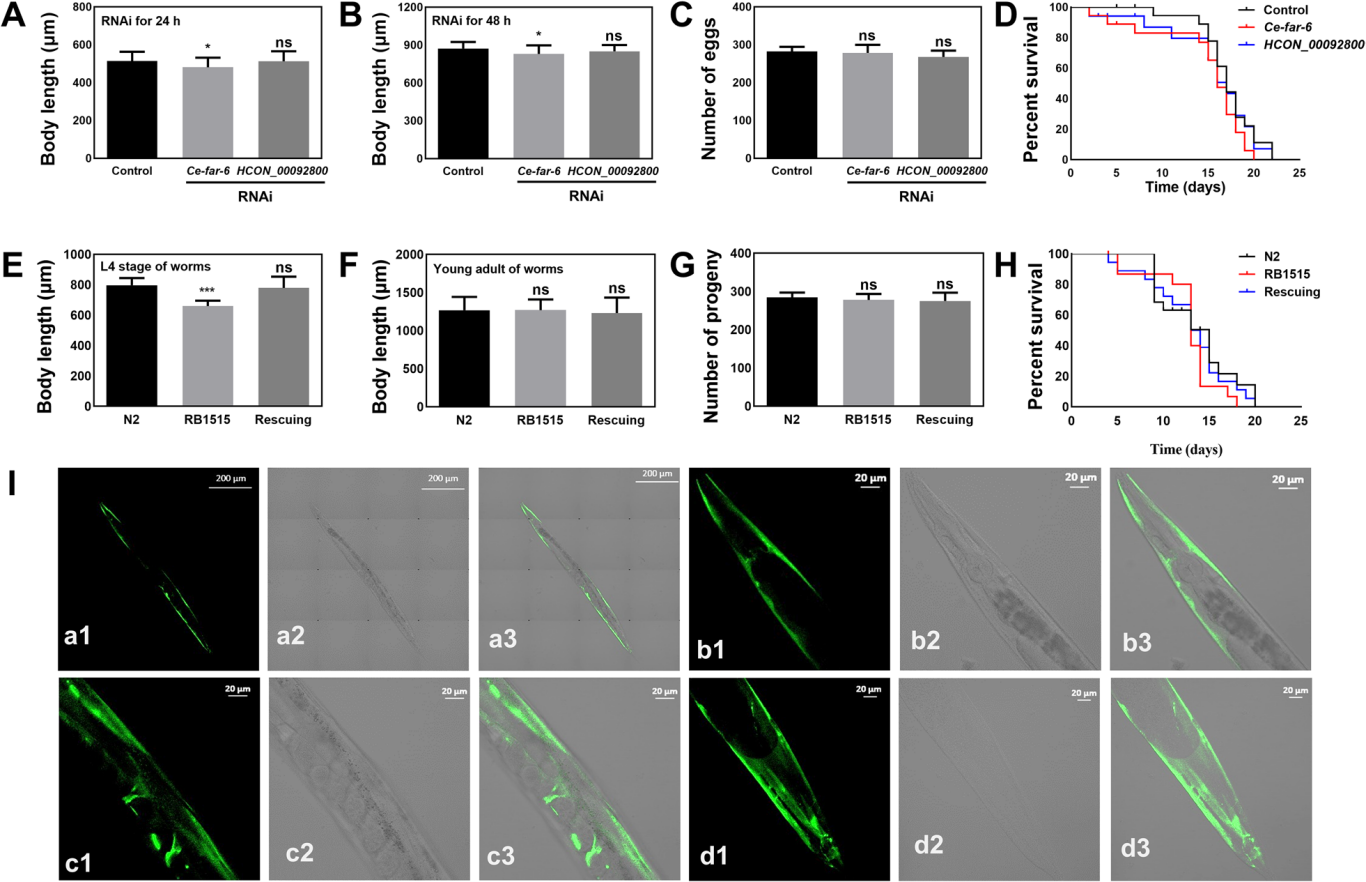
Effect of heterologous RNA interference (RNAi) or overexpression of *Ce-far-6* on the development, reproduction, and survival of *Caenorhabditis elegans* and heterologous expression of HCON_00092800. **A–D** Body length, number of eggs, and lifespan of *C. elegans* after *HCON_00092800* sequence mediated RNAi. *Ce-far-6* and *cry1Ac* are used as positive and irrelative controls, respectively. **E–H** Body length, number of eggs, and lifespan of wildtype (N2), *far-6* mutant (RB1515), and HCON_00092800 rescuing *C. elegans*. Error bars are the mean ± standard deviation (SD). ***P < 0.001, *P < 0.05, ns = not significant. **I** Heterologous expression of HCON_00092800 driven by the promoter of *Ce-far-6* in RB1515 strain of *C. elegans*. The overall, head, middle, and tail views of heterologous expression are indicated in subpanels **a**, **b**, **c**, and **d**, respectively. Numbers 1, 2, and 3 represent green fluorescence, differential interference contrast (DIC), and merge channels, respectively. Scale bars: 200 μm or 20 μm as indicated

Indeed, RNAi of *Ce-far-6* resulted in a slight but significant (*P* < 0.05) decreased body length (about 480 µm) after 24 h compared with negative control (about 520 µm; Fig. 4A). Decreased body length (about 800 µm) was also observed after 48 h of RNAi treatment compared with negative control (about 900 µm) (Fig. 4B). In addition, *Ce-far-6* RNAi did not influence the number of eggs produced by and lifespan of *C. elegans* (Fig. 4C, D). Full-length dsRNA of *HCON_00092800* mediated significant (*P* < 0.01) gene knockdown (heterologous RNAi) of *Ce-far-6* in *C. elegans* (Additional file 4: Fig. S2B), but surprisingly did not result in obvious phenotypes such as shorted body length, number of progeny, or lifespan of *C. elegans* (Fig. 4A–D).

Compared with the N2 strain of *C. elegans*, RB1515 exhibited significantly (*P* < 0.001) decreased body length after 24 h of culturing (Fig. 4E). This *Ce-far-6* mutant body length phenotype was completely rescued by heterologous expression of *HCON_00092800* in the RB1515 strain (Fig. 4E). Interestingly, the *Ce-far-6* mutant strain did not exhibit significantly shortened body length after 48 h of culturing, and expression of *HCON_00092800* did not influence the body length of treated worms (Fig. 4F). No significant difference was observed in aspects of the number of progeny or lifespan between wildtype and RB1515 strains (Fig. 4G, H). *Ce-far-6* appeared to control the body length of early-stage larvae in *C. elegans*. *HCON_00092800* is clearly a functional orthologue of the *Ce-far-6* and therefore was named *Hc-far-6* (Under GenBank accession no. OQ862326).

### Different tissue expression of FAR-6 between *C. elegans* and *H. contortus*

Using an enhanced green fluorescence protein indicator, the activity of *Ce-far-6* promoter (P_*Ce-far-6*_) was revealed predominantly in the hypodermis and weakly in the pharynx and anal area of the first generation produced by injected worms (Additional file 4: Fig. S2C). Determined by the activity of *C. elegans* P_*Ce-far-6*_, heterologous *Hc-far-6* was also found to be transcribed and then protein expressed in the hypodermis of offspring produced by treated worms (Fig. 4I). However, the 2000-bp sequence upstream *Hc-far-6* did not work in the free-living *C. elegans*, limiting the understanding of gene transcription and protein expression of this gene in parasitic *H. contortus*.

**Fig. 5:**
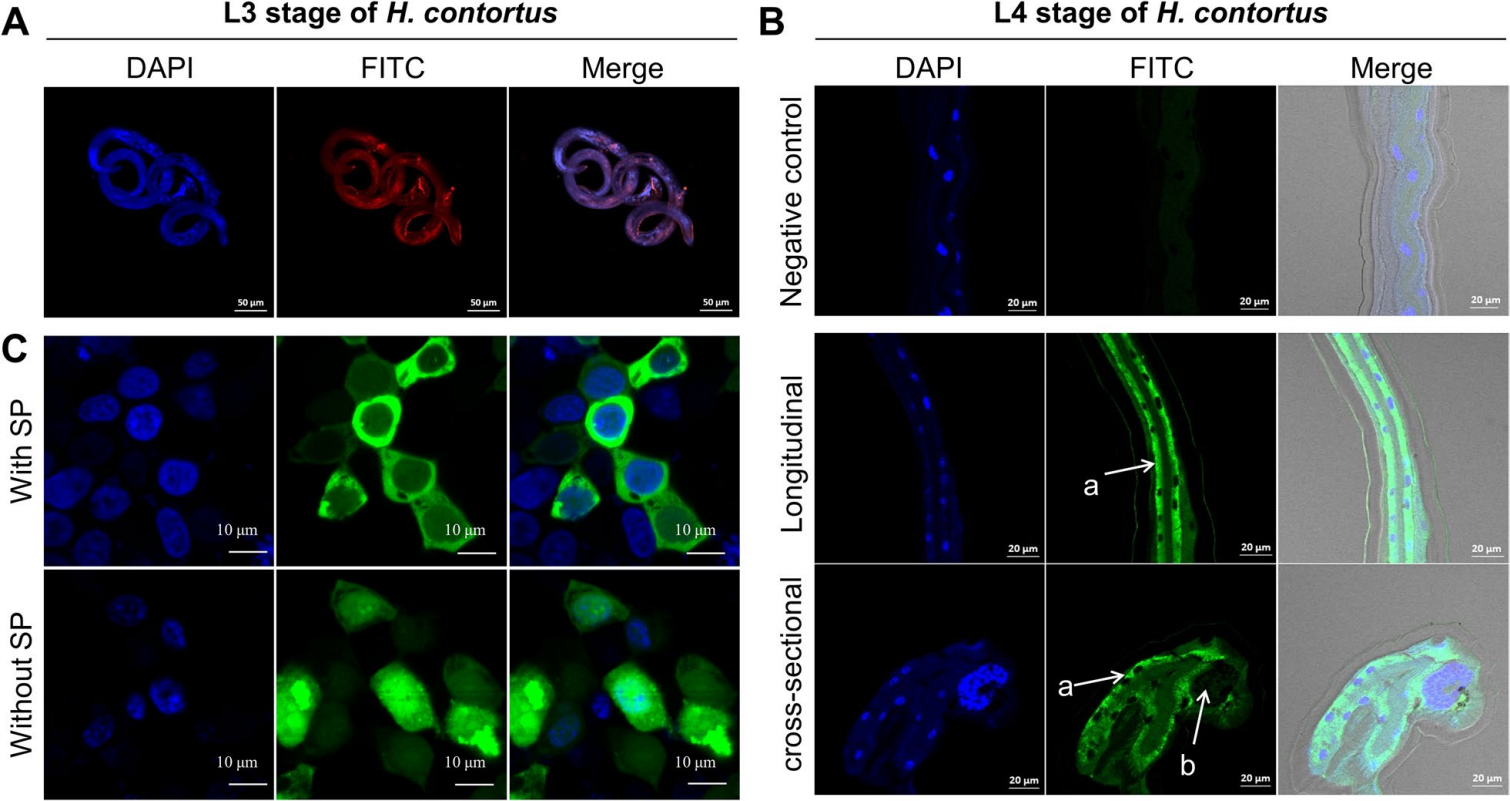
Tissue localisation of *Hc*-FAR-6 in *Haemonchus contortus*. **A** Indirect immunofluorescence of *Hc*-FAR-6 in the infective third larval (L3) stage of *H. contortus*. Mouse anti-r*Hc*-FAR-6 is used as the primary antibody and Alexa Fluor^™^ Plus 647 (red) is employed as the second antibody. **B** Indirect immunofluorescence of *Hc*-FAR-6 in the fourth larval (L4) stage of *H. contortus*. Mouse anti-r*Hc*-FAR-6 is used as the primary antibody and Alexa Fluor^™^ Plus 488 (green) is employed as the second antibody. The pre-immune serum is employed as a negative control. Lowercase letter 'a' indicates the intestine and 'b' indicates the gonad of *H. contortus*. DAPI, 4ʹ,6-diamidino-2-phenylindole. FITC, fluorescein isothiocyanate. Scale bars: 50 μm, 20 μm, or 10 μm as indicated. **C** Subcellular distribution of *Hc*-FAR-6 with/without signal peptide in human embryonic kidney 293 (HEK 293 T) cells. SP, signal peptide

A polyclonal antibody-based indirect immunofluorescence assay was employed to unveil the tissue expression pattern of *Hc*-FAR-6 in *H. contortus*. In the infective larvae of *H. contortus* (which are similar in size to the adult *C. elegans*), no distinct expression pattern was observed for *Hc*-FAR-6, faintly discernible in the hypodermis, musculature, and intestine (Fig. 5A). By contrast, due to the highest transcriptional level of *Hc-far-6* in the L4 stage of *H. contortus*, protein expression of *Hc*-FAR-6 was predominantly observed in the intestine of this early parasitic stage (Fig. 5B). Weak signals were also observed at the cuticle of both the infective and parasitic stages of *H. contortus* (Fig. 5A, B). Given that *Hc-far-6* encodes a functional signal peptide-containing protein in vivo (Fig. 5C), *Hc*-FAR-6 is likely to be secreted by intestinal cells and play roles in parasitism at the early stage of nematode infection in the host.

## Discussion

As fatty acid- and retinol-binding proteins (FARs) are exclusively identified in nematodes and not predicted in mammals, these proteins represent potential targets against nematode infection. Using a comparative approach, three *far* genes that might play a role in the parasitic stage of barber’s pole worm were identified; one of the three genes was elucidated as a functional orthologue of *far-6* in the model organism *C. elegans*; different from the predominant protein expression of FAR-6 in the hypodermis of free-living nematodes, FAR-6 was found mainly expressed in the intestine of *H. contortus* and is likely involved in parasitism at the early stage of nematode infection.

There is a mostly conserved FAR family in *C. elegans* and *H. contortus*. Although FARs are thought to be nematode-specific lipid-binding proteins, which have been comprehensively identified in nematodes of clades III, IV, and V, no homologues were predicted in clade I species [26]. This recent finding suggests a genus-level divergence of the *far* family among nematodes. In the current study, by integrating and manually curating previous work [26, 31, 32], 12 *far* gene homologues were identified in the parasitic nematode *H. contortus* (clade V), which is comparable to the number of *far* genes in the free-living *C. elegans* (*n* = 10; clade V). Based on the phylogeny of these genes, tandem duplication was found in both *C. elegans* and *H. contortus*, resulting in many-to-many orthologues between the two species. For instance, a cluster of *HCON_00092770*, *HCON_00092780*, *HCON_00092790*, *HCON_00092800*, and *HCON_00092810* genes in *H. contortus* showed close relationships with *far-1*, *far-2*, and *far-6* in *C. elegans*. Particularly, *HCON_00092780*, *HCON_00092790*, and *HCON_00092800* exhibited similar transcriptional alterations to those of *far-2* and *far-6* among developmental stages, indicating their involvement in similar biological processes.

Unlike *far-2* gene, *far-6* did not control nematode fat content. Nematodes have lost the ability to synthesise lipids de novo, and their need for fatty acids (required for collagen synthesis, cuticle construction and embryonic/post-embryonic development) and retinol (required for spermatozoa generation, tissue differentiation and immune-related responses) is only satisfied externally [15, 20, 23, 55, 56]. However, either free fatty acids or free retinoids are relatively insoluble in water, susceptible to oxidation, and potentially toxic to nematodes [19, 44]. FARs represent one of the approaches evolved by nematodes to acquire and transport free fatty acids and retinol [19, 26, 44, 57, 58]. Therefore, FAR protein-coding genes (e.g. *far-2*) have been linked to fat content in nematodes [25]. However, in the current study, RNAi activated by the sequence of *Ce-far-6* and heterologous *HCON_00092800* or overexpression of HCON_00092800 protein did not affect the content of either total fatty acids or randomly selected fatty acids, determined by different methods, in *C. elegans*. This result might be explained by the low sequence identity among *Ce-far-2*, *Ce-far-6*, and *HCON_00092800* genes (Additional file 3: Fig. S1), or by the functional compensation of other paralogues, but suggests that the two paralogues might play different roles in *C. elegans* [43].

Indeed, RNAi of *Ce-far-6* in *C. elegans*, which was verified in a *Ce-far-6* mutant strain, resulted in a shortened body length. Surprisingly, this *Ce-far-6* mutant-associated phenotype was completely rescued by *HCON_00092800*, which was therefore formally named *Hc-far-6*. However, this phenotype disappeared at the adult stage of *C. elegans*, which might result from decreased efficacy of RNAi, but is more likely a functional compensation of *Ce-far-1*, which has been associated with a similar phenotype (i.e. dumpy) [24]. By contrast, apart from the involvement in body-length control at the larval stage, a lower transcriptional level or even deficiency of *Ce-far-6* did not affect the progeny and lifespan of *C. elegans* [59, 60]. We did not test whether *Hc-far-6* plays a role in controlling the body length of larvae in *H. contortus*, as its encoded protein exhibited a totally different tissue localisation from that in *C. elegans*. In the free-living nematode, the active promoter of *Ce-far-6* in the hypodermis determines the tissue protein expression of heterologous *Hc*-FAR-6. However, in the parasitic L4 stage of *H. contortus*, *Hc*-FAR-6 was mainly detected in the intestine, suggesting a distinct role of this gene in parasitism within host animals.

FARs are predicted secretory proteins and have actually been detected in the excretory-secretory products of parasitic nematodes, including *H. contortus* [61]. For instance, a recombinant nematode FAR (i.e. HCON_00092780) has been shown to functionally inhibit host transforming growth factor-β signalling and the expression of associated cytokines [30], suggesting a role in parasite-host interactions. In particular, FARs of *Steinernema carpocapsae* (an insect parasitic nematode) have been shown to modulate host immunity, causing an increase in susceptibility to bacterial co-infection [31]. In addition, several *far* genes exhibited a male-dominant transcription in *H. contortus*, which is likely associated with the involvement of retinol in spermatogenesis, based on information from mammals [62, 63]. A systematic nomenclature rule for *far* genes [26, 43] and efficient reverse genetic tools for parasitic nematodes [64–66] would promote the functional understanding of *far* genes in nematode lipid biology and their potential as interaction targets against nematode infections.

## Conclusions

We manually curated the fatty acid- and retinol-binding protein coding genes in the barber’s pole worm and elucidated that *HCON_00092800* is a functional orthologue of *far-6* in *C. elegans*, which does not control worm fatty acid content but plays a role in regulating larval body length of *C. elegans* and is probably involved in lipid acquisition-associated parasitism. A better understanding of *far* genes in nematode biology should pave the way to a novel intervention strategy to control nematode infection and associated diseases in humans and animals.

## Supplementary Information

**Additional file 1: Table S1.** Primer sets used for protein expression and RNA interference experiments.

**Additional file 2: Table S2.** Identification of fatty acid-/retinol-binding protein coding genes in *Haemonchus* *contortus*.

**Additional file 3: Fig. S1.** Pair-wise sequence comparison between *HCON_00092800 *and *Ce-far-1*, *Ce*-*far-2* and *Ce-far-6. *Gene sequences of *Caenorhabditis elegans* are downloaded from WormBase (https://www.wormbase.org) through accession nos. F02A9.2 (*Ce-far-1*), F02A9.3 (*Ce-far-2*) and W02A2.2 (*Ce-far-6*). Alignments were performed using the EMBOSS Needle (https://www.ebi.ac.uk/Tools/psa/emboss_needle/). Sequences in red and blue indicate primers for sequence amplification used in *HCON_00092800*- and *Ce-far-6*-mediated RNAi, respectively.

**Additional file 4: Fig. S2.** Heterologous expression of HCON_00092800 and heterologous RNA interference (RNAi) of *Ce-far-6* in *Caenorhabditis elegans*. Western blot analyses for overexpression of HCON_00092800 in N2 (lane 1), heterologous expression of HCON_00092800 in RB1515 (lane 2), and *Ce*-FAR-6 in RB1515 (lane 3). M, protein ladder. **B** Relative mRNA levels of the *Ce-far-6* gene in the *Ce-far-6* or the *HCON_00092800* sequence mediated-RNAi worms. The *cry1Ac *is used as an irrelative control. Error bars are presented as mean ± standard error of the mean (SEM). **P < 0.01, ***P < 0.001. **C** Activity of *Ce-far-6* promoter in *C. elegans*. GFP, green fluorescence protein; *DIC* differential interference contrast. Scale bars: 200 μm or 20 μm as indicated.

## Data Availability

All data are included within the manuscript and the corresponding figures and tables. The sequence of *HCON_00092800* (*Hc-far-6*) was submitted to the GenBank database under accession no. OQ862326.
