# Supplementary material for: Fatty acid- and retinol-binding protein 6 does not control worm fatty acid content in Caenorhabditis elegans but might play a role in Haemonchus contortus parasitism
Source: Parasit Vectors. 2023 Jul 10;16:230. doi: 10.1186/s13071-023-05836-8 (PMC10334587; doi:10.1186/s13071-023-05836-8)
Supplement: Supplementary file 1 — Additional file 1: Table S1. Primer sets used for protein expression and RNA interference experiments. [file 13071_2023_5836_MOESM1_ESM.docx]

**Additional file 1: Table S1. Primer sets used for protein expression and RNA interference experiments**

| **Primer ID** | **Purpose** | **Primer sequence 5'-3'** |
| --- | --- | --- |
| *Hc-far-2.2a 1F* | CDS amplification | ATGGTCCGTTTCGCCATTCC |
| *Hc-far-2.2a 1R* | CDS amplification | GTTCAAGGGAAGGGCAGCAAGCT |
| *Hc-far-2.2a* qF | qPCR | TTGCTGGATTCACCAACATACC |
| *Hc-far-2.2a* qR | qPCR | CCTGTCGGATTCAGAAAGACC |
| *Hc-tub* qF | qPCR | TGTTCCATCACCCAAGGTATCC |
| *Hc-tub* qR | qPCR | TGACAGACACAAGGTGGTTGAGAT |
| *Hc-far-2.2a 2F* | Prokaryotic expression | CGAGCTCCTAGTTCAAGGGAAGGGCAGCAAGC |
| *Hc-far-2.2a 2R* | Prokaryotic expression | CGGGGTACCATGGTCCGTTTCGCCATTCCAAT |
| *Ce-far-6* Q F | qPCR | TCCAATTTCACGCTTACCACA |
| *Ce-far-6* Q R | qPCR | CCTTAATAGCAGCTTTCTCCTCA |
| *Ce-actin-1* F | qPCR | GGA ATG TGC AAG GCC GGAT |
| *Ce-actin-1* R | qPCR | ACC TCT CTT GGA TTG GGCCTC |
| *Ce-far-6* 3F | RNAi | TCCCCGCGGATGATCCGCATCTTCCTTGTC |
| *Ce-far-6* 3R | RNAi | CGGGGTACCGTAGTTCACGATCTGTTGGA |
| *Hc-far-2.2a* 3F | RNAi | TCCCCGCGGATGGTCCGTTTCGCCATTCC |
| *Hc-far-2.2a* 3R | RNAi | CGGGGTACCGTTCAAGGGAAGGGCAGCAAGCT |
| *Ce-far-6*-Prom F | Promoter amplification | AACTGCAG ATTTCAATGAAAATTTATGTGC |
| *Ce-far-6*-Prom R | Promoter amplification | TGCTCTAGATGCTATAACATTTTAAATTATATAG |
| *Hc-far-2.2a*-Prom F | Promoter amplification | AACTGCAG TTCGCCAGCCGAAATTAATCAC |
| *Hc-far-2.2a*-Prom R | Promoter amplification | TGCTCTAGAGACTGAAAATTGCAGGTCGATTTCT |
| *Hc-far-2.2a* 4F | Heterologous expression | CGCGGATCCATGGTCCGTTTCGCCATTCCAAT |
| *Hc-far-2.2a* 4R | Heterologous expression | CGGGGTACCTTGTTCAAGGGAAGGGCAGCAAGC |

Note: The restriction sites are underlined
