# Supplementary material for: Fatty acid- and retinol-binding protein 6 does not control worm fatty acid content in Caenorhabditis elegans but might play a role in Haemonchus contortus parasitism
Source: Parasit Vectors. 2023 Jul 10;16:230. doi: 10.1186/s13071-023-05836-8 (PMC10334587; doi:10.1186/s13071-023-05836-8)
Supplement: Supplementary file 2 — Additional file 2: Table S2. Identification of fatty acid-/retinol-binding protein coding genes in Haemonchus contortus. [file 13071_2023_5836_MOESM2_ESM.docx]

**Additional file 2: Table S2. Identification of fatty acid-/retinol-binding protein coding genes in *Haemonchus contortus***

| **WormBase ParaSite (Version: WBPS17)** | **Previously known as (gene names*)** | **Previous accession number** |
| --- | --- | --- |
| HCON_00092810 | FAR-1_Hco | HCOI00909100.t1 |
|  | FAR-2_Hco | HCOI00378700.t1 |
|  | *Hc-far-1(1)* | / |
|  | *Hc-far-1(2)* | / |
|  | *Hc-far-1(3)* | / |
|  | *Hc-far-1(4)* | / |
| HCON_00092770 | FAR-3_Hco | HCOI00908700.t1 |
|  | FAR-4_Hco | HCOI00500200.t1 |
|  | *Hc-far-4* | / |
| HCON_00092780 | FAR-5_Hco | HCOI00908800.t1 |
|  | FAR-6_Hco | HCOI00500300.t1 |
|  | *Hc-far-3(1)* | / |
|  | *Hc-far-3(2)* | / |
| HCON_00092790 | FAR-7_Hco | HCOI01356900.t1 |
|  | FAR-8_Hco | HCOI00908900.t1 |
|  | *Hc-far-2(1)* | / |
|  | *Hc-far-2(2)* | / |
|  | *Hc-far-2(3)* | / |
|  | *Hc-far-2(4)* | / |
| HCON_00092800 | FAR-9_Hco | HCOI00909000.t1 |
|  | FAR-10_Hco | HCOI01356800.t1 |
| HCON_00089630 | FAR-11_Hco | HCOI00607700.t1 |
|  | FAR-12_Hco | HCOI00614900.t1 |
| HCON_00093410 | FAR-13_Hco | HCOI00700800.t1 |
|  | FAR-14_Hco | HCOI00206800.t1 |
|  | *Hc-far-5(1)* | / |
| HCON_00093190 | FAR-15_Hco | HCOI02099100.t1 |
|  | FAR-16_Hco | HCOI00494100.t1 |
| HCON_00120470 | FAR-17_Hco | HCOI00678000.t1 |
| HCON_00042410 | FAR-18_Hco | HCOI01362000.t1 |
|  | FAR-19_Hco | HCOI00490300.t1 |
|  | *Hc-far-6* | / |
| HCON_00093170 | / | / |
| HCON_00109090 | / | / |

/ means not found; ***** gene names were given by Kuang et al. or Yuan et al. [26, 32].
