# Supplementary figures and images for: Fatty acid- and retinol-binding protein 6 does not control worm fatty acid content in Caenorhabditis elegans but might play a role in Haemonchus contortus parasitism
Source: Parasit Vectors. 2023 Jul 10;16:230. doi: 10.1186/s13071-023-05836-8 (PMC10334587; doi:10.1186/s13071-023-05836-8)

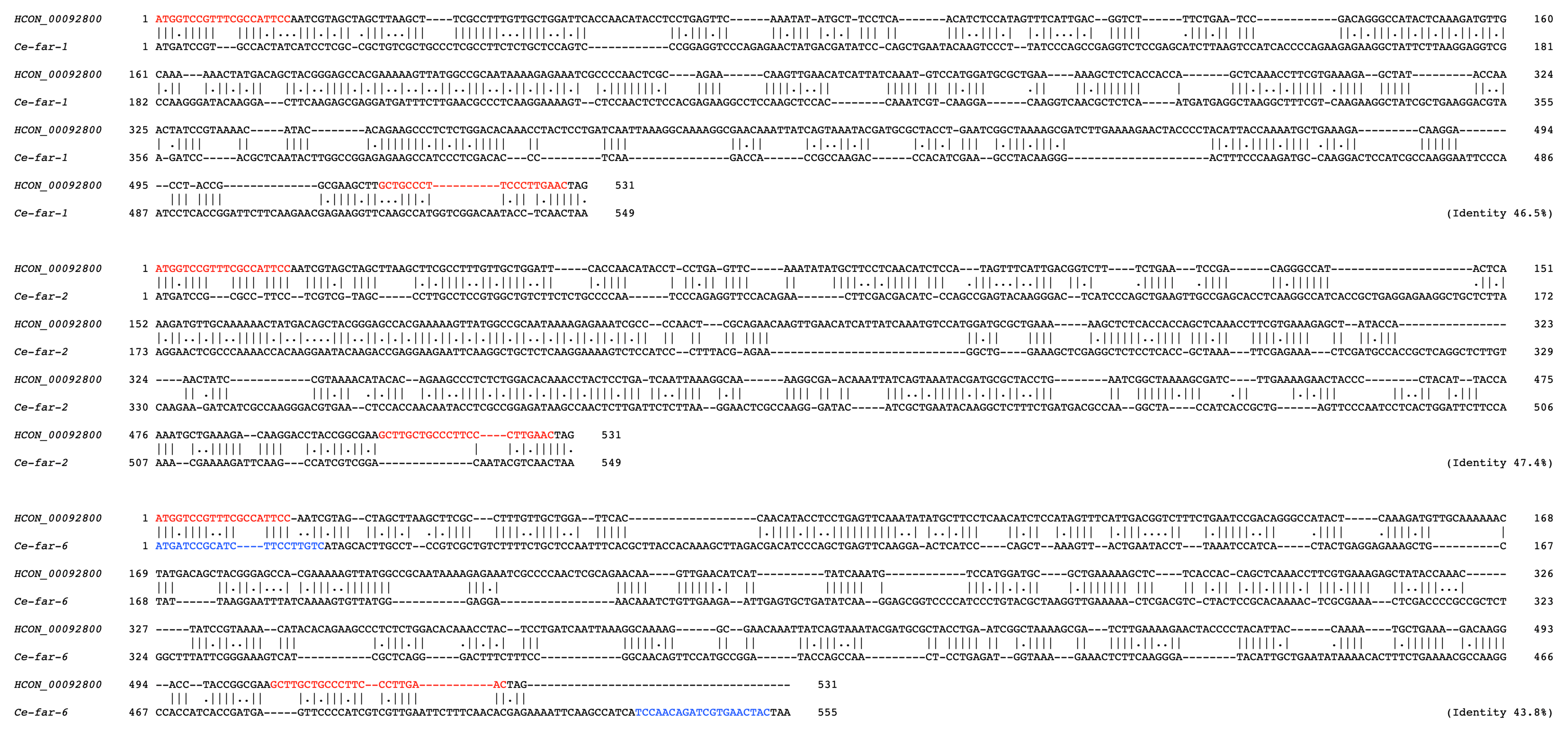

Supplement: Supplementary file 3 — Additional file 3: Fig. S1. Pair-wise sequence comparison between HCON_00092800 and Ce-far-1, Ce-far-2 and Ce-far-6. Gene sequences of Caenorhabditis elegans are downloaded from WormBase (https://www.wormbase.org) through accession nos. F02A9.2 (Ce-far-1), F02A9.3 (Ce-far-2) and W02A2.2 (Ce-far-6). Alignments were performed using the EMBOSS Needle (https://www.ebi.ac.uk/Tools/psa/emboss_needle/). Sequences in red and blue indicate primers for sequence amplification used in HCON_00092800- and Ce-far-6-mediated RNAi, respectively. [file 13071_2023_5836_MOESM3_ESM.tif]

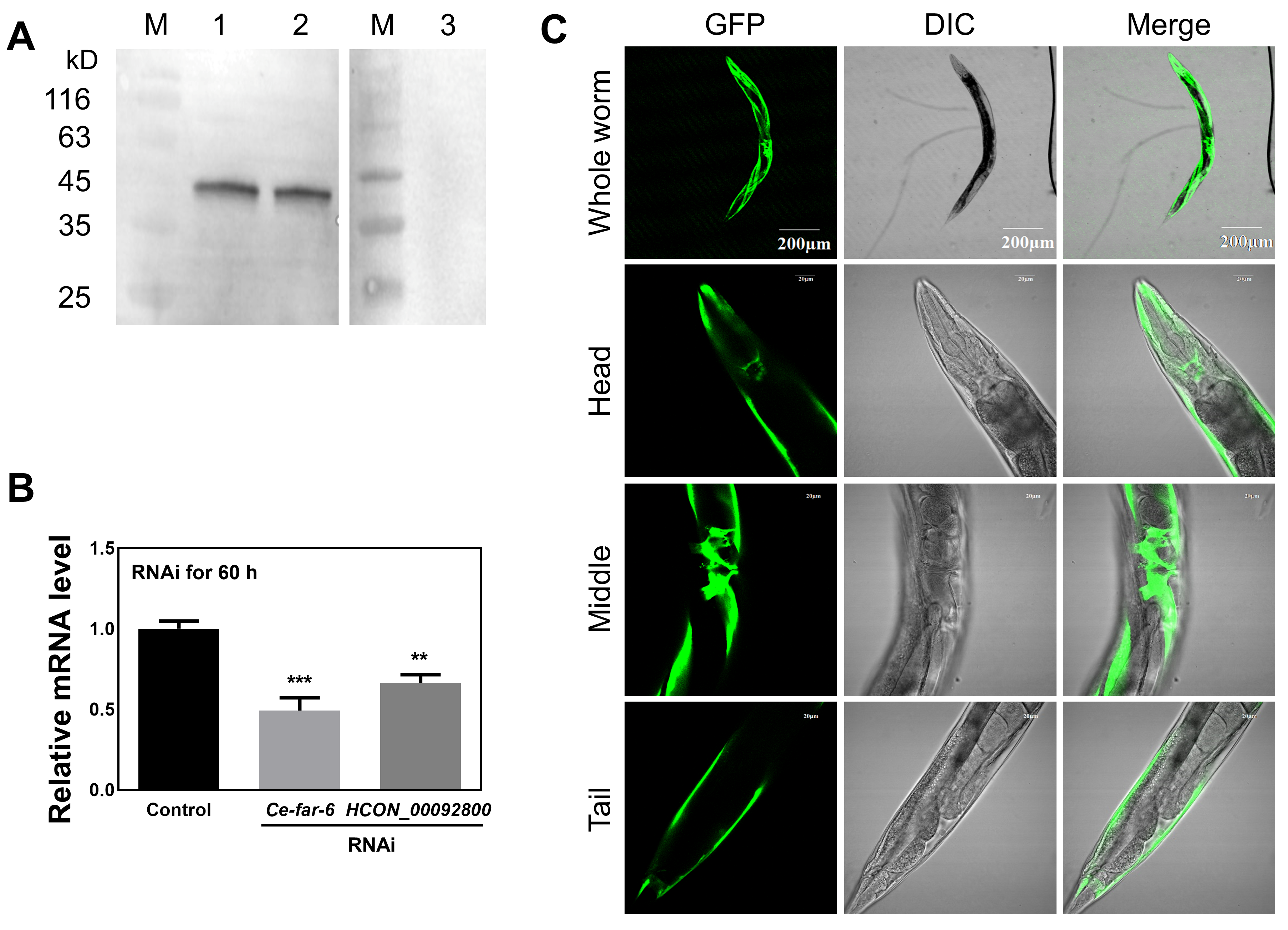

Supplement: Supplementary file 4 — Additional file 4: Fig. S2. Heterologous expression of HCON_00092800 and heterologous RNA interference (RNAi) of Ce-far-6 in Caenorhabditis elegans. Western blot analyses for overexpression of HCON_00092800 in N2 (lane 1), heterologous expression of HCON_00092800 in RB1515 (lane 2), and Ce-FAR-6 in RB1515 (lane 3). M, protein ladder. B Relative mRNA levels of the Ce-far-6 gene in the Ce-far-6 or the HCON_00092800 sequence mediated-RNAi worms. The cry1Ac is used as an irrelative control. Error bars are presented as mean ± standard error of the mean (SEM). **P < 0.01, ***P < 0.001. C Activity of Ce-far-6 promoter in C. elegans. GFP, green fluorescence protein; DIC differential interference contrast. Scale bars: 200 μm or 20 μm as indicated. [file 13071_2023_5836_MOESM4_ESM.tif]
